# T-Cell Lymphoma Presenting with Auricular and Parotid Gland Involvement

**DOI:** 10.4274/tjh.2015.0217

**Published:** 2016-02-17

**Authors:** Birgül Öneç, Alper Koç, Elif Nisa Ünlü, İlhan Ünlü, Hüseyin Yaman, Durdu Mehmet Köş

**Affiliations:** 1 Düzce University Faculty of Medicine, Department of Hematology, Düzce, Turkey; 2 Düzce University Faculty of Medicine, Department of Internal Medicine, Düzce, Turkey; 3 Düzce University Faculty of Medicine, Department of Radiology, Düzce, Turkey; 4 Düzce University Faculty of Medicine, Department of Otorhinolaryngology, Düzce, Turkey

**Keywords:** Parotid gland, T-Cell lymphoma, Auricula

## TO THE EDITOR

The external auditory canal is an unusual presenting site for lymphomas, with only a few case reports in the literature [1,2]. Malignant lymphomas arising from the salivary glands are also uncommon, accounting for approximately 5% of extranodal lymphomas, and the majority of them are of B-cell lineage. Primary salivary gland T-cell lymphomas are extremely rare [3,4,5].

**Figure 1:**
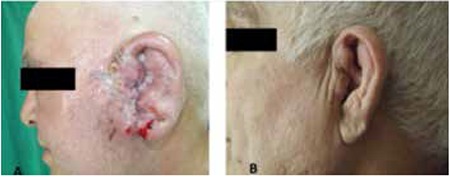
A) A suppurative lesion that consisted of ulcerated areas and granulation tissue was observed in the external auditory canal and preauricular region. B) Significant regression of lesion after 4 cycles of CHOP (cyclophosphamide, doxorubicin, vincristine, prednisone) treatment.

A 63-year-old man was admitted with swelling of the left side of his face and left auricle. Considered as an infection, it was empirically treated with systemic and topical antibiotics. Examination by an otorhinolaryngologist revealed a suppurative lesion that consisted of ulcerated areas and granulation tissue in the external auditory canal and preauricular region in addition to edema (Figure 1A). A lobulated mass lesion of 47x39 mm arising from the left parotid and extending to the left auditory canal was detected in computed tomography (CT) with accompanying lymph nodes in the left subauricular and cervical localization. Fine-needle biopsy was nondiagnostic and incisional biopsy found only severely active chronic inflammation. Finally, deep excisional biopsy revealed CD3-, CD5-, and CD30-positive and S100-negative lymphoid cells diffusely infiltrating the dermis. The case was considered as stage 2 peripheral T-cell lymphoma not otherwise specified peripheral T-cell lymphoma-not otherwise specified (PTCL-NOS) and CHOP (cyclophosphamide, doxorubicin, vincristine, prednisone) protocol was started. Despite clinical improvement in the lesions (Figure 1B), CT revealed progression after the fourth cycle. The second-line treatment is ongoing with DHAP (dexamethasone, high-dose ara-C, cisplatin) at the 8th month of follow-up.

The auricula, external auditory canal, and parotid glands are unusual locations for T-cell lymphoma. Presentations of lymphomas are indistinguishable from other swellings of the auricle or parotid gland and therefore a high index of suspicion should be maintained in patients who present with presumptive cutaneous infections that do not respond to antibiotic therapy in these locations. A suppurative auricular lesion suggests an infectious disease rather than a lymphoma, but primary cutaneous lymphomas and cutaneous manifestations of lymphomas must be kept in mind. Early excisional biopsy may prevent excessive waste of time with unnecessary antibiotherapies. Parotid lymphomas are most likely to be B-cell non-Hodgkin lymphoma, but non-B-cell lymphomas have a more aggressive course in all salivary gland lymphomas. Both B-cell and T-cell lymphomas share many morphological similarities; therefore, immunohistochemical analysis is required for proper assignment of lineage of salivary gland lymphomas [5]. Reactive lymphoid infiltrate located in the periphery of the lymphoma may contribute to the delay of diagnosis and larger biopsy samples are needed.
